# Single-molecule localization microscopy imaging of extracellular vesicle DNA in recipient cells

**DOI:** 10.1186/s12967-025-07563-3

**Published:** 2026-01-03

**Authors:** Xingfu Zhu, Venkatesh Kumar Chetty, Jamal Ghanam, Anisa Hila, Qiqi Yang, Hilmar Strickfaden, Mischa Bonn, Christoph Cremer, Peter F. Hoyer, Xiaomin Liu, Basant Kumar Thakur

**Affiliations:** 1 Department of Molecular Spectroscopy, Max Planck Institute for Polymer Research, Ackermannweg 10, 55128 Mainz, Germany; 2 Department of Pediatrics III, University Hospital Essen, Hufelandstrasse 55, 45147 Essen, Germany; 3 Department of Gastroenterology, Hepatology and Transplant Medicine, Medical Faculty, University Hospital Essen, Hufelandstrasse 55, 45147 Essen, Germany; 4 Department of General, Visceral, Vascular and Transplant Surgery, Medical Faculty, University Hospital Essen, Hufelandstrasse 55, 45147 Essen, Germany; 5 Cell Imaging Centre, Faculty of Medicine and Dentistry, B-120 Katz Group Centre, University of Alberta, Edmonton, AB T6G 2T9 Canada; 6 Institute of Molecular Biology (IMB), Ackermannweg 4, 55128 Mainz, Germany; 7 Department of Pediatrics II, University Hospital Essen, Hufelandstrasse 55, 45147 Essen, Germany; 8 European Liquid Biopsy Society (ELBS), Hamburg, Germany

**Keywords:** Small extracellular vesicles, EV-DNA, Single-molecule localization microscopy, SMLM, BODIPY

## Abstract

**Background:**

Small extracellular vesicles (sEVs) are critical mediators of intercellular communication in both physiological and pathological contexts, including cancer, by transporting key biomolecules between cells. However, the biogenesis, packaging, and functional roles of DNA associated with sEVs (EV-DNA) remain poorly understood, largely due to the lack of efficient EV-DNA labeling dyes compatible with super-resolution imaging techniques.

**Results:**

Here, we employed BODIPY, a green-emitting, buffer-independent blinking fluorophore, to label EV-DNA cargo and applied single-molecule localization microscopy (SMLM) for the first time under physiological conditions to achieve nanoscale imaging of EV-DNA in recipient cells. This approach overcomes conventional fluorophore limitations, enabling high-resolution dual-color imaging without quenching artifacts. We further developed a co-labeling strategy combining click chemistry (EV-DNA) and nanobody-based immunostaining (CD63-GFP⁺-sEVs), achieving precise multi-target labeling with a calculated linkage error of ~ 2 nm. Dual-color SMLM imaging revealed limited co-localization between EV-DNA and CD63-GFP⁺-sEVs, suggesting that EV-DNA may associate with distinct vesicle populations. Additionally, dual-color SMLM combined with cluster analysis indicated partial spatial proximity between EV-DNA and the cytoplasmic DNA sensor cyclic GMP-AMP synthase (cGAS), suggesting potential but limited functional interactions.

**Conclusions:**

The SMLM-based imaging approach established in this study provides a powerful platform for investigating the packaging and subcellular fate of EV-DNA at nanometer resolution. Our results uncover new aspects of EV-DNA biology, including limited association with CD63⁺ vesicles and partial proximity to cGAS, suggesting alternative intracellular pathways. This versatile approach will enable detailed exploration of EV-DNA dynamics and its functional roles in health and disease.

**Supplementary Information:**

The online version contains supplementary material available at 10.1186/s12967-025-07563-3.

## Graphical Abstract

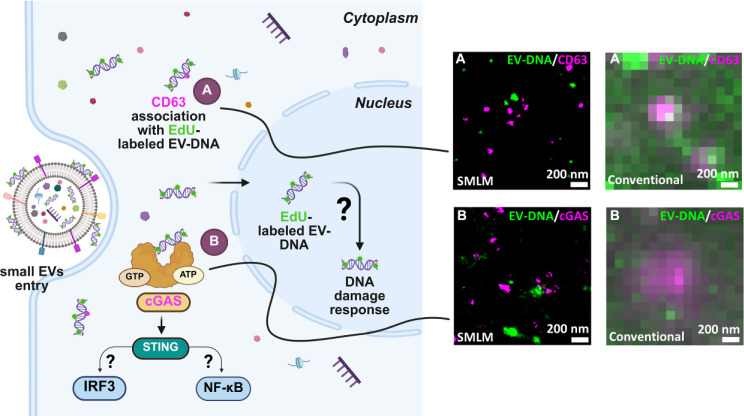

## Background

Small extracellular vesicles (sEVs) are lipid bilayer-enclosed vesicles that are less than 200 nm in diameter and are secreted by both healthy and malignant cells released across the extracellular space [1–3]. sEVs contain various molecular cargoes, including proteins, lipids, and nucleic acids (DNA, mRNA, miRNA, etc.), which reflect the parental cells from which they originate [4–6]. These sEV cargoes play functional roles in various diseases, including cancer and neurodegenerative disorders, by mediating intercellular communication [7–9].

Compared to other sEV cargoes, the biological function of DNA associated with sEVs (EV-DNA) in health and disease is not well understood [10]. This is because EV-DNA functional studies to date have used only fluorescence confocal imaging, which has a maximum spatial resolution of ~ 250 nm leading to an imprecise explanation for the interaction of EV-DNA with various subcellular components in the recipient cells [11–20]. Therefore, it is highly desirable to implement super-resolution imaging to reveal the fate and localization of EV-DNA within recipient cells.

Single-molecule localization microscopy (SMLM) is one of the super-resolution imaging techniques that utilizes photoactivatable/ blinking fluorophores to obtain images with very high spatial resolutions, typically around 20 nm [21, 22]. Although SMLM has been used in several EV studies for nanoscale imaging of single vesicles or their cargoes, its application to EV-DNA interactions in recipient cells has been limited. One of the significant hurdles is the absence of dyes or probes that are both efficient in labeling EV-DNA and compatible with other fluorophores used in SMLM. Additionally, most of the currently used photo-switching fluorophores, including the gold standard Alexa Fluor 647, require specialized imaging buffers. In contrast, other fluorophores may exhibit suboptimal blinking properties under the same buffer conditions, thereby interfering with multi-color SMLM imaging [23–25].

For instance, when we previously applied dual-color SMLM to image non-transferred green fluorescent protein (GFP)^+^-labeled sEVs containing 5-ethynyl-2’-deoxyuridine (EdU)-tagged-EV-DNA, we observed substantial quenching of the GFP signal [11]. This was due to a combination of GFP’s poor blinking behavior under SMLM conditions and the copper-catalyzed click chemistry used for EdU detection with Alexa Fluor 647, which generates reactive copper species that can damage the GFP chromophore and reduce its fluorescence [11]. These limitations underscore the need for the development of buffer-independent blinking fluorophores that are compatible with Alexa Fluor 647 [26], thereby enabling efficient dual-color SMLM imaging to visualize EV-DNA interactions with subcellular structures in recipient cells at nanometer-scale resolution.

Photoblinking fluorophores are essential for achieving super-resolution in SMLM. In this study, we employed a green-emitting, buffer-independent blinking fluorophore, boron-dipyrromethene (BODIPY), for nanoscale imaging of EV-DNA. BODIPY dyes are known for their synthetic versatility, high fluorescence quantum yield, excellent photostability, and superior biocompatibility, making them highly suitable for biological imaging applications [27–30]. Importantly, BODIPY has been previously shown to exhibit favorable blinking behavior in the same imaging buffer used for Alexa Fluor 647, enabling effective dual-color SMLM imaging [31]. We therefore employed BODIPY for SMLM imaging of EV-DNA.

Here, we utilized BODIPY-azide to label EdU-tagged EV-DNA via click chemistry, allowing for the first-time detection of EV-DNA in recipient cells using SMLM under physiological conditions in PBS. In addition, we developed a co-labeling strategy that combines click chemistry for EV-DNA with nanobody-based immunostaining of CD63-GFP⁺ sEVs, enabling high-precision dual-color SMLM with a calculated linkage error (~ 2 nm) compared with conventional antibody labeling (~ 20 nm). This approach allowed visualization of the spatial association between EV-DNA and CD63⁺ vesicles within recipient cells at nanometer resolution. Functionally significant, we also demonstrated the capability of dual-color SMLM imaging to resolve the proximity between EV-DNA and the cytoplasmic DNA sensor cyclic GMP-AMP synthase (cGAS), a key mediator of virus-induced inflammation and cancer metastasis [32].

Overall, our study demonstrates that dual-color SMLM using BODIPY in PBS provides a reliable platform for mapping EV-DNA uptake, intracellular trafficking, and subcellular interactions at nanometer resolution. Our results reveal previously unrecognized aspects of EV-DNA biology, including very low association with CD63⁺ vesicles and partial proximity to cGAS, suggesting alternative intracellular pathways. This versatile imaging strategy is highly adaptable for studying multiple EV biomarkers and offers a robust tool to investigate EV-DNA function in both physiological and pathological contexts.

## Materials and methods

### Cell culture

HEK293T, HEK293T-CD63-eGFP, HeLa, and U2OS cells were cultured in Dulbecco’s Modified Eagle’s Medium (DMEM) (Gibco, Paisley, UK) containing 10% fetal bovine serum (FBS) and 1% Penicillin/ Streptomycin (P/S) in a humidified incubator at 37 °C and 5% CO_2_. HEK293T-CD63-eGFP is a gift from Prof. Bernd Giebel, University Hospital Essen, Germany. It is a stable cell line, in which the canonical marker CD63 is overexpressed and labeled with eGFP.

### Isolation and characterization of small EVs

For the isolation of small extracellular vesicles (sEVs), HEK293T and HEK293T-CD63-eGFP cells were cultured in 8 × 145 cm^2^ dishes containing 25 ml of DMEM media prepared using 10% EV-depleted FBS and 1% P/S and cultured for 72 h at 37 °C and 5% CO_2_. EV-depleted FBS was obtained using an ultracentrifugation device by centrifuging FBS at 100,000x g for 18 h. After culturing cells for 72 h, cell conditioned media (CCM) were further processed by centrifuging them at 500x g for 10 min and 3000x g for 20 min (4 °C) to remove cell debris and apoptotic bodies. Following this, as previously explained by Chetty et al. 2022 [11], sEVs were isolated from 0.2 μm filtered CCM using Tangential Flow Filtration (TFF- Easy; Hansa Biomed, Tallin, Estonia), Size Exclusion Chromatography (qEV10 column; IZON Science, Christchurch, NZ) and Ultrafiltration (Amicon Ultra-4 10 kDa centrifugal filter, Merck Millipore, Darmstadt, Germany), collectively known as TSU. We have previously determined that small extracellular vesicles are more efficiently fractionated and enriched in fractions 2 and 3 without apoptotic bodies, when TSU-based isolation is performed [11]. Additionally, we evaluated that apolipoprotein contamination was lower in these sEV fractions (F2 and F3), which qualifies them for sEV-based functional studies [11]. In this regard, we mixed these two fractions and used them for further characterization and functional experiments.

As previously described [11], HEK293T sEVs were characterized according to MISEV2023 guidelines using nanoparticle tracking analyzer (NTA), transmission electron microscopy (TEM), and western blot. NTA was performed to measure the number of particles per ml and the particle average size present in the HEK293T sEV preparation. Negative staining of HEK293T sEVs was done with phosphotungstic acid, and the stained sEVs were visualized in TEM at the Electron Microscopy Unit (EMU) of the Imaging Center Essen (IMCES), University Hospital Essen. TEM images (16-bit) were processed using EMMENU image software (Version 4.09.83). Next, semi-dry western blot was performed with HEK293T cells and their corresponding sEVs using EV canonical markers (Rabbit-α-TSG101, Sigma, Cat. No: HPA006161; Rabbit-α-Hsp70, System Biosciences, Cat. No: EXOAB-Hsp70A-1; Mouse-α-CD81, Biolegend, Cat. No: 349502) and cellular or EV-negative marker (Rabbit-α-Calnexin, Abcam, Cat. No: ab22595).

### Extraction of EV-DNA prior to and following dsDNase treatment

To assess whether DNA is located on the outer membrane or inside the EVs, 100 µL of HEK293T sEVs were pre-treated with 6 µL of dsDNase (ThermoFisher Scientific, Waltham, MA, USA) and 10X dsDNase buffer at 37 °C for 5 min. The reaction was halted by adding 0.5 M EDTA. Subsequently, DNA was extracted from both intact sEVs and the dsDNase-treated sEVs using the QIAamp DNA Micro Kit (Qiagen, Hilden, Germany). To determine the presence of single-stranded DNA inside the sEVs, 3 µL of the extracted HEK293T EV-DNA was subjected to a second round of dsDNase treatment under the same conditions. Finally, 5 µL aliquots of the four EV-DNA sample sets, along with genomic DNA, were loaded onto a 2% agarose gel and electrophoresed at 100 V for 1 h. The gel was then stained with SYBR Safe Gold nucleic acid dye (ThermoFisher Scientific, Waltham, MA, USA) for 30 min before imaging.

### EdU labeling using BODIPY-azide

To fluorescently tag EV-DNA in the recipient cells, EdU (5-ethynyl-2’-deoxyuridine) was used to metabolically label EV-DNA in HEK293T and HEK293T-CD63-eGFP donor cells, and the corresponding sEVs were isolated. At day 0, ~ 5000 HeLa cells were seeded into an 8-well chambered coverslip with glass bottom (#80807, ibidi) and incubated overnight at 37 °C and 5% CO_2_. The next day, they were treated with 500,000 HEK293T or HEK293T-CD63-eGFP sEVs containing EdU-labeled EV-DNA for 3 h. As a positive control, HeLa cells were incubated with 20 µM F-ara-EdU for 3 h. For the negative control, HeLa cells were treated with 500,000 HEK293T sEVs containing no EV-DNA-EdU label. After 3 h of treatment, the cells were fixed with 4% paraformaldehyde (PFA) in PBS for 10 min, permeabilized with 0.1% Triton X-100 in PBS for 10 min, and then blocked with 3% BSA in PBS for 1 h. Following blocking, EV-DNA-EdU was labeled using Click-it Plus EdU Alexa Fluor 488 kit (C10637, Thermofisher Scientific) following the manufacturer’s instructions. Instead of Alexa Fluor 488-azide, 5 µM BODIPY-azide (21430, Lumiprobe, Germany), which is commercially available without further chemical modification, was used. The cells were then washed three times with PBS for 5 min and stained with DAPI, followed by one wash with PBS. For SMLM imaging, DAPI was not used. Afterwards, they were stored in PBS at 4 °C in a refrigerator until imaging.

### Labeling of CD63-GFP^+^-sEVs using Alexa Fluor 647 -anti-GFP nanobody

HeLa cells were incubated with CD63-GFP⁺ sEVs at a ratio of 50:1 (sEV particle number/recipient cells) for 3 h under the same conditions described in the previous section. Following incubation, the same protocols were used for fixation, permeabilization, and blocking. After blocking, internalized CD63-GFP⁺-sEVs were labeled with Alexa Fluor 647 -conjugated anti-GFP nanobody (Alexa Fluor 647 -Nb; 50 nM; #gb2AF647, Proteintech) in PBS containing 1% BSA and 0.1% saponin for 1 h at RT. Cells were then washed three times with PBS for 5 min each, counterstained with DAPI, and rinsed once more with PBS. After staining, samples were stored at 4 °C until imaging.

### Co-labelling of EV-DNA-EdU and CD63-GFP^+^-sEVs

To evaluate the compatibility of co-labeling EV-DNA-EdU with BODIPY-azide and CD63-GFP⁺ sEVs using Alexa Fluor 647 -conjugated anti-GFP nanobody (Alexa Fluor 647 -Nb), we employed U2OS-Nup96-GFP cells, which express GFP fused to the nuclear pore complex protein Nup96. Three experimental conditions were tested to assess whether copper ions interfere with the GFP signal. As a positive control, untreated cells were imaged without any labeling. In the second condition, Nup96-GFP was labeled with Alexa Fluor 647 -Nb (50 nM) in PBS containing 1% BSA and 0.1% saponin for 1 h at RT. In the third condition, cells were pre-treated with 1 mM CuSO₄ for 30 min at RT before labeling Nup96-GFP with Alexa Fluor 647 -Nb, to assess the effect of copper on nanobody binding and GFP signal.

In parallel, U2OS-Nup96-GFP cells were incubated with 20 µM F-ara-EdU for 3 h. After fixation, permeabilization, and blocking as described above, Nup96-GFP was labeled again using Alexa Fluor 647 -Nb under the same conditions. The cells were then washed three times with PBS (5 min each), followed by a post-fixation step with 4% PFA at RT for 10 min. After a final PBS wash, incorporated F-ara-EdU was labeled with BODIPY-azide (green) following the same procedure as mentioned previously.

Once optimal co-labeling conditions for EdU (BODIPY-azide) and GFP (Alexa Fluor 647 -Nb) were confirmed in U2OS cells, HeLa cells were incubated with HEK293T-derived CD63-GFP⁺-sEVs containing EV-DNA-EdU at a ratio of 50:1 (sEV particles per recipient cell), for 3 h. Following fixation, permeabilization, and blocking as described previously, CD63-GFP⁺ sEVs were first labeled using Alexa Fluor 647 -Nb. As with U2OS cells, a second fixation with 4% PFA was performed prior to EV-DNA-EdU labeling using BODIPY-azide.

### Immobilization of HEK293T-CD63-GFP^+^-sEVs containing EV-DNA-EdU

An 8-well chambered coverslip with glass bottom (#80807, ibidi) was used for immobilizing sEVs. Chambers were first washed once with PBS, then incubated with 2% Hellmanex (9-307-011-4-507, Hellma) for 1 h, followed by three PBS washes. Subsequently, chambers were treated with 1 M KOH for 1 h and rinsed once more with PBS. Next, diluted HEK293T-CD63-GFP⁺-sEVs containing EV-DNA-EdU (1:100 in PBS) were added to the chambers and incubated overnight at 4 °C to allow immobilization.

The following day, surfaces were rinsed three times with PBS to remove unbound sEVs. CD63-GFP⁺-sEVs were labeled with Alexa Fluor 647 -conjugated anti-GFP nanobody (A647-Nb, 1:200 dilution in PBS) for 1 h at RT, followed by three washes with PBS (5 min each). The surfaces were then fixed with 4% PFA and 0.2% glutaraldehyde in PBS for 10 min at RT, then washed once with PBS. Finally, EV-DNA-EdU was labeled via click chemistry using BODIPY-azide for 30 min at RT, followed by three PBS washes (5 min each).

### Interaction of EV-DNA with cGAS in HeLa cells

HeLa cells were incubated with HEK293T sEVs containing EV-DNA-EdU at a ratio of 50:1 (sEV particle number/recipient cells). Then, the cells were fixed, permeabilized, and blocked with a blocking buffer following the same protocol. cGAS was then labeled by incubating the cells with rabbit anti-MB21D1 primary antibody (1:200 dilution, #HPA031700, Sigma) for 1 h at RT. After washing with PBS, cells were incubated with Alexa Fluor 647 -conjugated goat anti-rabbit secondary antibody (ab150083, Abcam) for 1 h at RT. Following a single PBS wash, the cells were post-fixed with 4% PFA at RT for 10 min. Finally, EV-DNA-EdU was labeled using BODIPY-azide according to the previously described protocol.

### Wide-field imaging

Images presented in Figs. 2B, 3B, 4B, 5A, and Supplementary Figure S1 were acquired using a Leica Thunder wide-field microscope. The system was equipped with 8 LEDs (395, 438, 475, 511, 555, 575, 635, and 730 nm) for illumination. Specific channels included 395 nm for DAPI, 475 nm for BODIPY and GFP, and 635 nm for Alexa Fluor 647.

### Super-resolution imaging

Super-resolution imaging was performed using a Leica SR GSD microscope equipped with an HCX PL APO 160 × 1.43 NA Oil CORR-TIRF objective lens and an EMCCD camera (iXon DU-897, Andor configured at 10 MHz readout speed, 14-bit depth, and 5.1× pre-amplification gain). Excitation lasers at 642 nm (500 mW) and 488 nm (300 mW) were used. For the 642 nm laser, an excitation filter (637–647 nm/400–410 nm), a dichroic beam splitter (637–647 nm/400–410 nm), and an emission filter (660–760 nm/449–451 nm) were employed. For the 488 nm laser, we used the excitation filter (483–493 nm/400–410 nm), dichroic beam splitter (483–493 nm/400–410 nm), and emission filter (505–605 nm/449–451 nm). Dual-band filters and beam splitters were selected to accommodate 405 nm back-pumping. Detailed imaging parameters for all single-color and dual-color SMLM experiments are provided in Table S1.

#### Reproducibility

For SMLM imaging of EV-DNA-EdU and CD63-GFP⁺-sEVs, a single cell was imaged and analyzed per session. To ensure reproducibility, three different cells were imaged and analyzed for each experiment (*n* = 3 cells per experiment). Consistent results were obtained across all replicates.

### Single-molecule localization microscopy (SMLM) image data analysis

All single-molecule localization microscopy (SMLM) movies were analyzed using the ThunderSTORM plugin in ImageJ [33]. The peak intensity threshold was set to 2.0 for EV-DNA-EdU, CD63-GFP⁺ sEVs, and cGAS datasets. Drift correction was applied using the cross-correlation method (3-bin setting) within ThunderSTORM. Continuous blinking events were merged and treated as a single molecule. Super-resolution image reconstruction was also performed in ThunderSTORM.

For SMLM images, a reconstruction pixel size of 5 nm was applied for all datasets. Localizations corresponding to EV-DNA-EdU clusters were subjected to Voronoi-based cluster analysis using SR-Tesseler [34], with the following default parameters: density factor = 2, minimum area = 2, minimum number of localizations = 5, maximum intensity = 10,000, and maximum number of localizations = 100,000. Spatial distance analysis between EV-DNA and cGAS was performed using the DiAna plugin in ImageJ (Fiji) [35], with the parameters radius- 2.0 and threshold- 20000.

### Data presentation

All graphs were generated using GraphPad Prism 10 (GraphPad Software, San Diego, CA, USA). The graphical illustration was created with BioRender.com, which was also used to align and format other figures for consistency [36].

## Results

### Small EVs and their associated DNA content characterization

As previously demonstrated [11], HEK293T small extracellular vesicles (sEVs) were isolated and enriched using the TSU method, which combines tangential flow filtration, size-exclusion chromatography, and ultrafiltration. These sEVs were characterized in accordance with the Minimal Information for Studies of Extracellular Vesicles 2023 (MISEV2023) guidelines, as recommended by the International Society for Extracellular Vesicles (ISEV) [3].

**Fig. 1:**
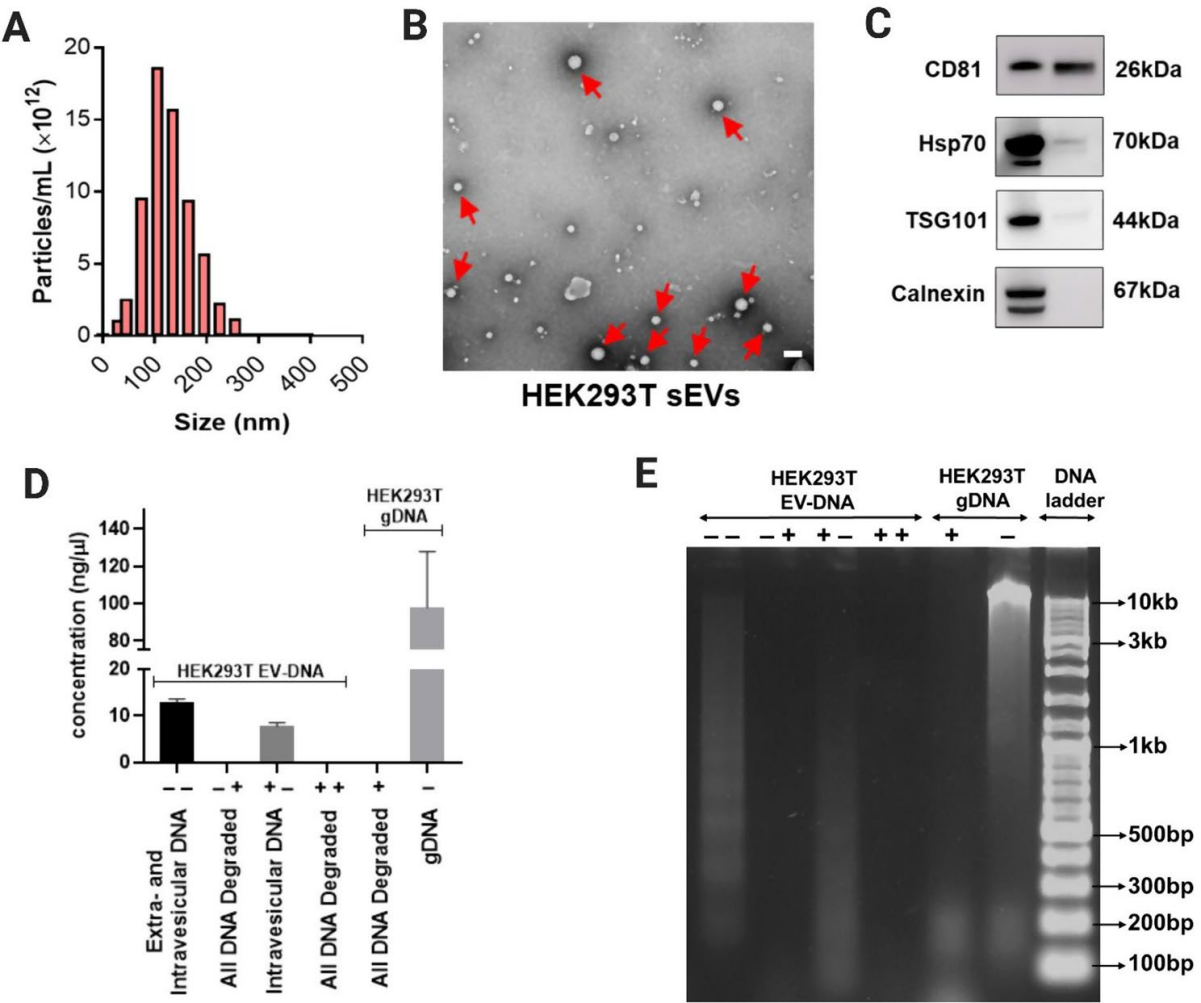
Characterization of small extracellular vesicles (sEVs) derived from HEK293T cells and associated DNA content (EV-DNA). (**A**) Nanoparticle tracking analysis (NTA) showing the size distribution and concentration of HEK293T sEVs. (**B**) Transmission electron microscopy (TEM) image of negative staining of HEK293T sEVs (6000X magnification). Red arrows indicate the presence of vesicle-like structures. Scale bar = 100 nm. (**C**) Western blot-based characterization of HEK293T sEVs using EV classical markers (CD81, Hsp70 and TSG101) and cell debris marker (calnexin). Assessment of extravesicular and intravesicular DNA in HEK293T sEVs following dsDNase treatment. HEK293T sEVs were subjected to single (±) or double (±, ++) treatments with dsDNase, an enzyme that digests double-stranded DNA, prior to DNA isolation. (**D**) DNA concentrations were quantified under four conditions: untreated (– –; includes both extra- and intravesicular DNA), single dsDNase-treated (+ –; intravesicular DNA only, and – +; all DNA degraded), double dsDNase-treated (+ +; all DNA degraded), and HEK293T genomic DNA (gDNA) as control. Data represent Mean ± S.D obtained from three independent experiments. (**E**). Agarose gel electrophoresis analysis showing the distribution of DNA fragments following each treatment. Single dsDNase digestion removed most DNA fragments in the ~ 2.5–10 kb range, indicating their extravesicular origin, whereas double digestion led to complete DNA loss, confirming that vesicle-associated DNA is entirely double-stranded and enclosed within the sEVs. DNA ladder is shown on the right with corresponding size markers

First, nanoparticle tracking analysis (NTA) was performed to quantify particle size distribution within the sEV preparation (Fig. 1A). NTA data showed that the majority of particles ranged between 50 and 200 nm in diameter, consistent with the typical sEV size. Transmission electron microscopy (TEM) of negatively stained HEK293T sEVs further confirmed the presence of vesicles exhibiting the characteristic cup-shaped morphology with intact membranes (Fig. 1B, red arrows). Then, western blot analysis demonstrated that HEK293T sEVs were indeed enriched with more canonical EV markers such as CD81, TSG101, and Hsp70. The absence of calnexin signal suggested minimal contamination from cellular debris (Fig. 1C).

To distinguish between extravesicular and intravesicular DNA, HEK293T sEVs were subjected to a series of dsDNase treatments prior to DNA isolation. dsDNase specifically digests double-stranded DNA, allowing selective removal of DNA exposed on the vesicle surface. Four treatment conditions were applied: single digestion without (–) or with dsDNase (+), and double digestion combinations (– –), (+ –), (– +), and (+ +). In principle, the (– –) condition retains both extravesicular and intravesicular DNA, whereas the (+ –) condition retains only DNA protected inside the vesicles. As a control, HEK293T genomic DNA (gDNA) was also treated with or without dsDNase before DNA isolation.

DNA concentrations were quantified using a Nanodrop 2000 C Spectrophotometer (Thermo Fisher Scientific), and the values are shown in Fig. 1D. Single dsDNase digestion (+ –), which selectively degrades external double-stranded DNA, led to the loss of most DNA fragments in the ~ 2.5–10 kb range, suggesting that these larger fragments are predominantly extravesicular (Fig. 1E). Consistent with our previous findings [11], double dsDNase digestion (+ +) resulted in complete DNA degradation, confirming that the DNA associated with sEVs is double-stranded and located inside the vesicles.

### EV-DNA detection in the recipient cells using BODIPY

**Fig. 2:**
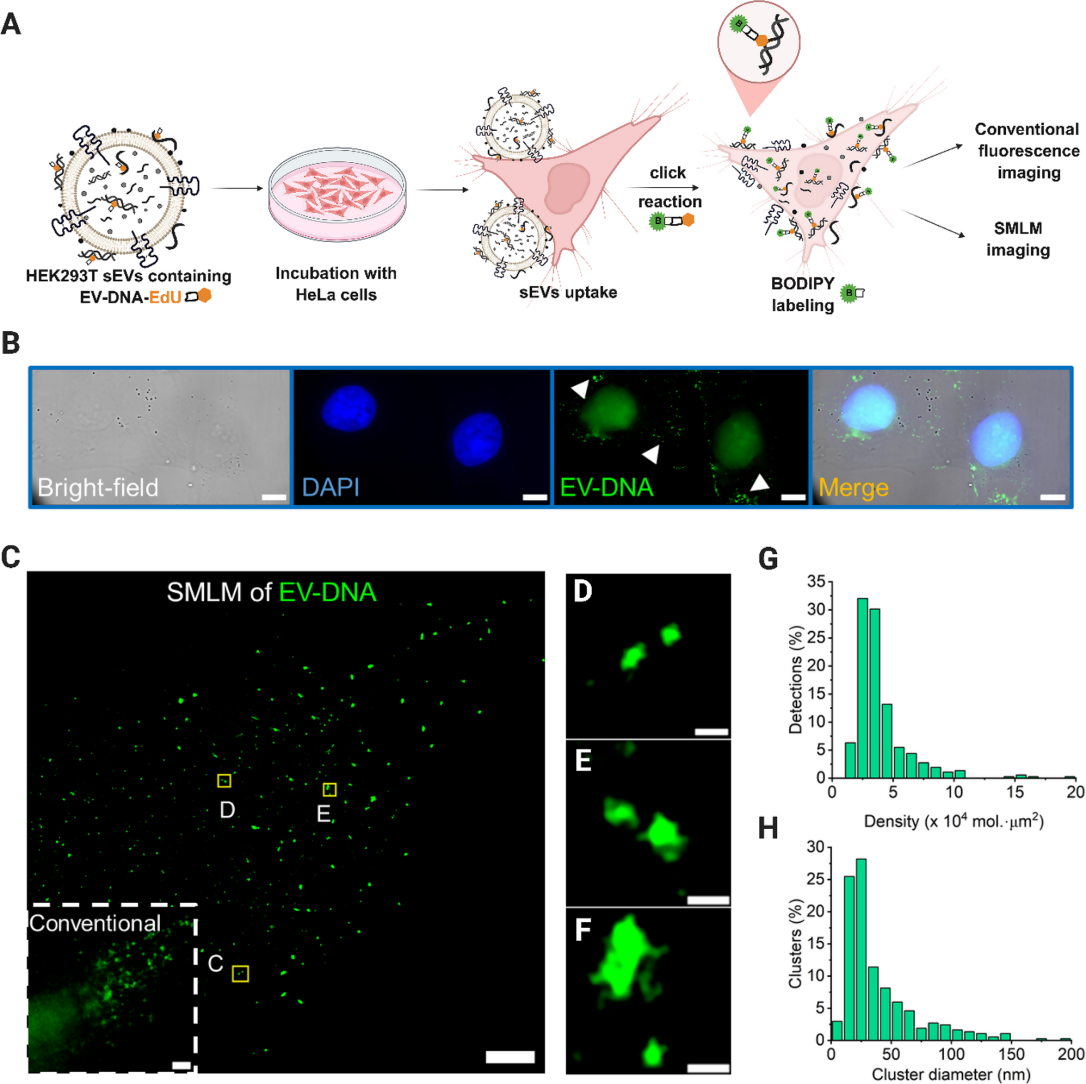
SMLM imaging of EV-DNA in the recipient cells. HeLa cells were incubated with HEK293T sEVs containing EV-DNA-EdU at a ratio of 50:1 (sEV particles per recipient cell). (**A**) Schematic illustration of the experimental workflow for imaging EV-DNA-EdU in recipient cells using BODIPY. (**B**) Conventional fluorescence images of HeLa recipient cells illustrating cell nuclei (DAPI, blue) and EV-DNA (BODIPY-azide, green). (**C**) SMLM imaging of HEK293T EV-DNA transferred into HeLa cells. The inset shown is the corresponding conventional wide-field image. (**D-F**) Magnifications of three different areas marked with yellow rectangles in **C**. (**G**) Distribution of the first-rank density (single-molecule localizations/µm^2^) of EV-DNA clusters in **C**. (**H**) Cluster size distribution for EV-DNA clusters in **C** (~ 360 clusters). Scale bars: 10 μm in **B**, 2 μm in **C**, 100 nm in **D** and **E**, and **F**

HeLa recipient cells were incubated for 3 h with HEK293T sEVs containing EdU-labeled EV-DNA at a ratio of 50:1 (sEV particle numbers per recipient cells), which was subsequently detected using BODIPY-azide via click chemistry (Fig. 2A). As previously demonstrated [11], these sEV preparations are free of unincorporated EdU, avoiding labeling artifacts. To confirm the specificity of BODIPY-azide labeling for EV-DNA-EdU, three control experiments were performed (Figure S1). First, HeLa cells incubated with free EdU for 3 h showed strong labeling of nascent DNA characterized by a high signal-to-noise ratio and specific nuclear targeting (top panel). As expected, only a subset of nuclei was labeled, reflecting the asynchronous nature of the cell population, as EdU is incorporated exclusively during the S phase. Second, untreated HeLa cells showed only minimal background (middle panel). Third, HeLa cells treated with sEVs lacking EdU-labeled DNA showed a negligible signal (bottom panel). The minimal background observed in untreated cells and the low positive signal in cells treated with sEVs lacking EdU-labeled DNA likely result from cellular autofluorescence and minor non-specific interactions of the click-labeling reagents [37, 38]. Together, these results confirm that BODIPY-azide specifically labels EV-DNA-EdU internalized by recipient cells, with minimal nonspecific background.

In line with our previous work, conventional fluorescence images demonstrated that EV-DNA-EdU was mainly located in the cytoplasm of HeLa cells after sEVs uptake (Fig. 2B) [11]. In addition, as previously found [39], we also observed nonspecific staining of BODIPY-azide in the nucleus (Fig. 2B). Nevertheless, as the primary focus of this study was to explore the interaction of EV-DNA with cytoplasmic biomolecules, potential nonspecific nuclear labeling by BODIPY-azide was not examined in detail. On the other hand, SMLM imaging of EV-DNA in HeLa cells in PBS buffer (Fig. 2C) enabled the visualization of EV-DNA clusters with a diameter smaller than 150 nm (Fig. 2[D-F]). Notably, SMLM resolved individual EV-DNA fragments as small as ~ 45 nm, which were indistinguishable by conventional microscopy (Fig. 2D). In addition, compared to other super-resolution imaging techniques, SMLM has unique advantages for quantifying the acquired data. To obtain the detailed quantification of EV-DNA clusters, we then performed the cluster analysis for the localization data sets with Voronoi segmentation methods [34] and calculated the local density of EV-DNA blinking events for each cluster (average 2.7 × 10^4^ single molecule signal/µm^2^) as well as the cluster size (average ~ 45 nm) (Fig. 2G, H). In general, cluster analysis revealed that the size of most EV-DNA fragments that were localized in the recipient cells is smaller than 50 nm. Nonetheless, we also found some EV-DNA clusters with diameters near 150 nm, which may be due to the aggregation or clustering of EV-DNA (Fig. 2F, H).

### Imaging CD63-GFP^+^-sEVs in the recipient cells using Alexa Fluor 647 -anti GFP nanobody

**Fig. 3:**
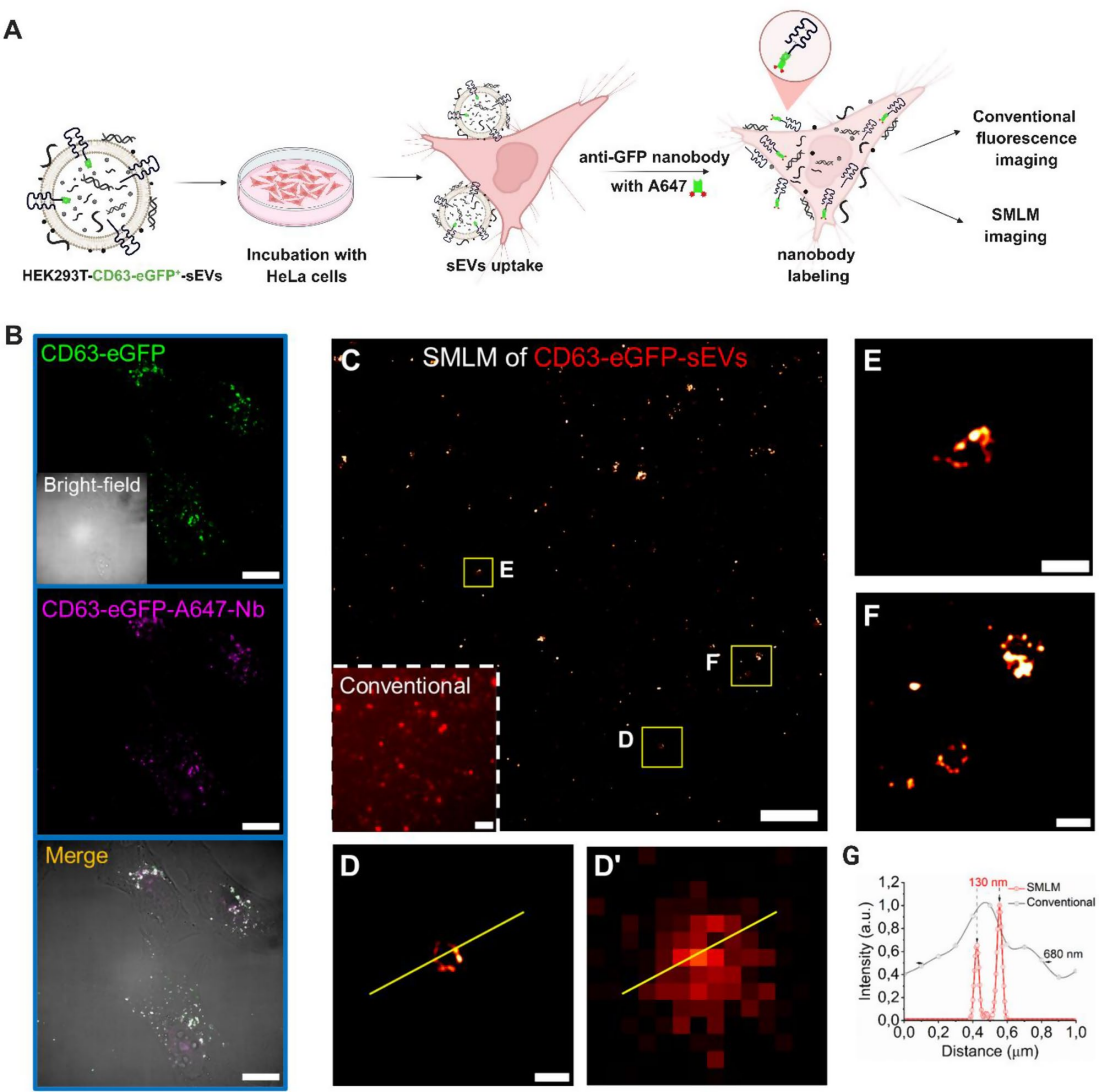
Imaging of HEK293T-CD63-eGFP^+^-sEVs using Alexa Fluor 647 -anti-GFP-nanobody. HeLa cells were treated with HEK293T-CD63-eGFP⁺-sEVs at a ratio of 50:1 (sEV particles per recipient cell) and subsequently stained with Alexa Fluor 647 -conjugated anti-GFP nanobody (Alexa Fluor 647 -Nb). (**A**) Schematic representation of the experimental workflow for visualization of HEK293T-CD63-eGFP^+^-sEVs in recipient cells using Alexa Fluor 647 -Nb. (**B**) Conventional fluorescence and bright-field images. Inset: the corresponding conventional wide-field image. (**C**) SMLM image. (**D-F**) Magnifications of three different areas marked with yellow rectangles in **C**. (**D**’) The corresponding conventional wide-field image of **D**. (**G**) Profiles of SMLM image and conventional wide-field image (yellow line shown in **D** and **D**’). Scale bars: 20 μm in **B**, 2 μm in **C**, and 200 nm in 3**D**-3**F**

To achieve the dual-color SMLM imaging of sEVs and EV-DNA cargo together in the next step, labeling and imaging sEVs need to be executed first. Although eGFP from HEK293T-CD63-eGFP^+^-sEVs can emit fluorescence, it was unsuitable for the SMLM imaging performed here; therefore, we used eGFP as the labeling site. HeLa cells were treated with CD63-eGFP⁺-sEVs at a ratio of 50:1 (sEV particles per recipient cell), and subsequently stained with Alexa Fluor 647 -conjugated anti-GFP nanobody (Alexa Fluor 647 -Nb) for conventional fluorescence and SMLM imaging (Fig. 3A). Dual-color images showed good colocalization of eGFP and Alexa Fluor 647 signals, confirming the successful labeling (Fig. 3B). Subsequently, SMLM imaging was then performed to detect CD63-eGFP^+^-sEVs on HeLa cells in a freshly prepared imaging buffer (Fig. 3C). In line with the study done by Hagey and coworkers [40], using SMLM imaging, we found that the structure of sEVs with a diameter of ~ 130 nm was clearly distinguishable in HeLa cells, while the conventional fluorescence image only showed a blurred image due to the diffraction limit (Fig. 3D, D’, G).

As sEVs are highly heterogeneous, we also observed different shapes of CD63-eGFP^+^-sEVs after cellular uptake (Fig. 3[D-F]). We can infer from Fig. 3D that CD63 tetraspanin is almost evenly distributed on the surface of sEVs, unlike the others (Figs. 3E, F). Similar to EV-DNA clusters in previous observations Fig. 2 (F, H), we also found clusters of CD63^+^-sEVs (Fig. 3E, F). sEV clusters are not unusual, as many studies, including ours, have previously shown sEV clusters within recipient cells using conventional light microscopy [11, 41, 42]. Taken together, it is evident that SMLM imaging could be potentially applied to visualize the morphology of individual sEVs after internalization in the recipient cells at the nanoscale level.

### Dual-color SMLM imaging of CD63^+^-sEVs and EV-DNA in the recipient cells

Dual-color SMLM was employed to simultaneously visualize EV-DNA cargo and CD63-eGFP⁺- sEVs in recipient cells. To validate the co-labeling strategy using click chemistry for EV-DNA (via BODIPY-azide) and immunofluorescence for GFP (via Alexa Fluor 647 -conjugated nanobody, Nb), we first utilized U2OS-Nup96-GFP cells, which express GFP at the nuclear pore complex. These cells served as a model system to test and optimize the labeling sequence. Initially, U2OS-Nup96-GFP cells were treated with or without copper ions prior to immunostaining with Alexa Fluor 647 -Nb. In the absence of copper, the GFP signal marking the nuclear pore complex precisely colocalized with Alexa Fluor 647 -Nb, confirming effective binding (Figure S2). However, in copper-treated cells, the GFP fluorescence was quenched, and Alexa Fluor 647 -Nb failed to specifically bind Nup96-GFP. These results indicated that copper ions not only quench GFP fluorescence but also interfere with the binding affinity of the nanobody toward GFP.

To overcome this, we modified the labeling sequence. After fixation, U2OS-Nup96-GFP cells pretreated with (2′S)-2′-Deoxy-2′-fluoro-5-ethynyluridine (F-ara-EdU) were first labeled with Alexa Fluor 647 -Nb. A second fixation step was then performed to preserve the Nup96-GFP–Alexa Fluor 647 -Nb complex, followed by BODIPY-azide click chemistry to label nascent DNA. This protocol produced robust dual signals for both nascent DNA (BODIPY) and Nup96-GFP (Alexa Fluor 647) in the nucleus, confirming the approach’s compatibility with dual-color SMLM (Figure S3).

**Fig. 4:**
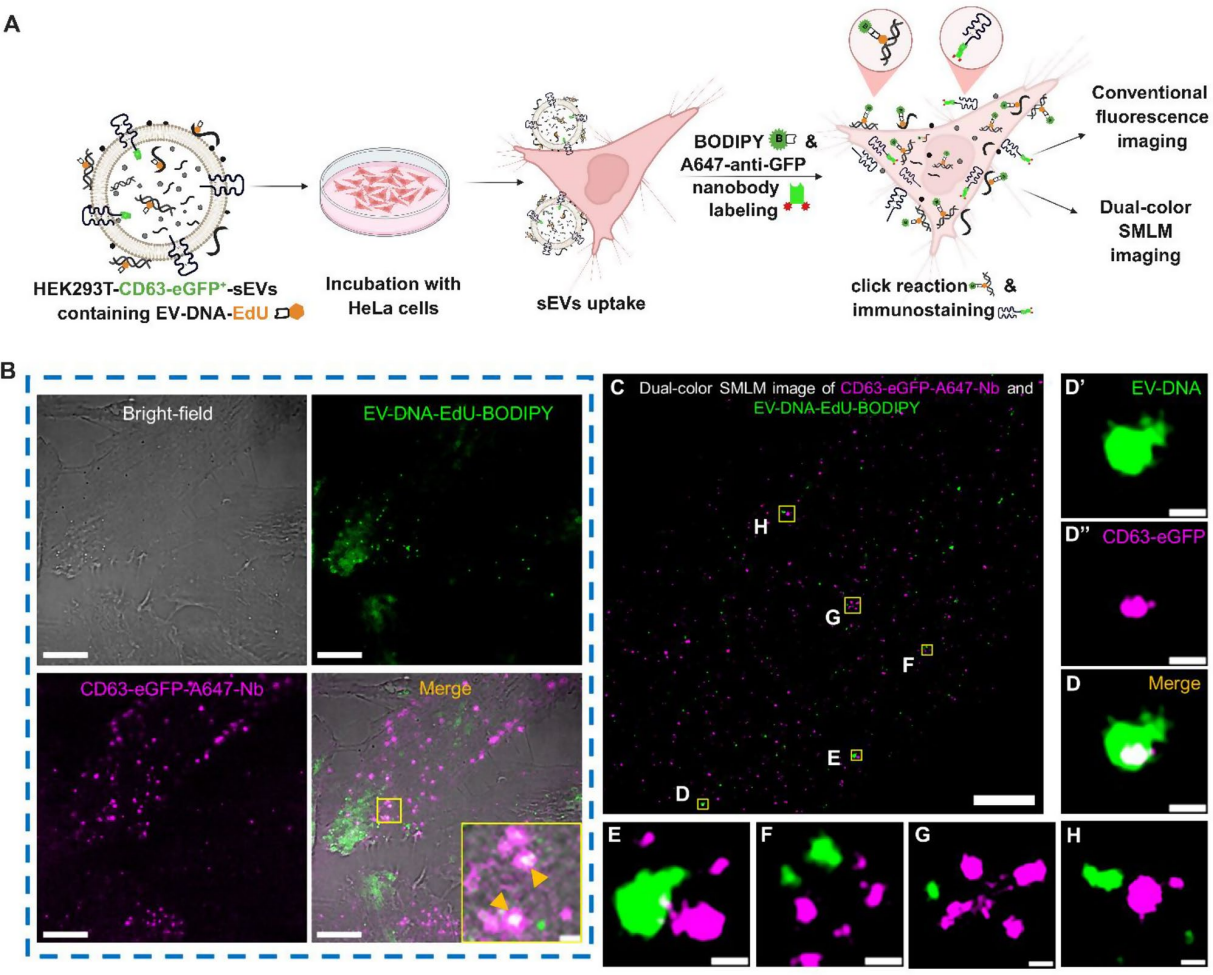
Colocalization of CD63^+^-sEVs and EV-DNA in the Recipient Cells. HeLa cells were treated with HEK293T-CD63-GFP^+^-sEVs containing EdU-labeled EV-DNA at a ratio of 50:1 (sEV particles per recipient cell). (**A**) Schematic illustration of the experimental strategy for dual labeling and super-resolution imaging of CD63^+^-sEVs and EV-DNA in recipient cells using Alexa Fluor 647 -conjugated anti-GFP nanobody (Alexa Fluor 647-Nb) and BODIPY-azide respectively. (**B**) Representative conventional wide-field fluorescence images showing partial colocalization (orange arrows) of CD63⁺-sEVs (purple, Alexa Fluor 647 -Nb) and EV-DNA (green, BODIPY-azide). (**C**) Reconstructed dual-color SMLM image of the same sample, providing nanoscale resolution. (**D**) Magnification of the yellow rectangle, **D**, shown in **C**, highlighting the clear colocalization of EV-DNA and CD63^+^-sEVs. (**E-H**) Magnification of four different areas marked with yellow rectangles (**E-H**) shown in **C**. Scale bars: 10 μm in **B**, 1 μm in **B** (inset), 2 μm in **C**, and 100 nm in **D**-**H**

We then applied the optimized protocol to HeLa cells incubated with CD63-GFP⁺-sEVs containing EdU-labeled EV-DNA. Cells were sequentially labeled with Alexa Fluor 647–conjugated anti-GFP Nb and BODIPY-azide via click chemistry to visualize CD63^+^-sEVs and EV-DNA, respectively (Fig. 4A). Conventional wide-field fluorescence imaging revealed only partial colocalization between EV-DNA and CD63⁺ sEVs (Fig. 4B), but the diffraction limit restricted resolution and interpretability. To address this limitation, we performed dual-color SMLM on the same sample. First, SMLM imaging of CD63-eGFP⁺-sEVs labeled with Alexa Fluor 647 -Nb was conducted under 642 nm laser illumination. Subsequently, EV-DNA labeled with BODIPY-azide was imaged under 488 nm illumination in the same buffer conditions. The reconstructed dual-color SMLM images (Fig. 4C) provided nanoscale resolution, eliminating the artifacts commonly observed in wide-field microscopy.

High-resolution SMLM imaging enabled us to determine unequivocally, without any typical artifacts common in conventional wide-field microscopy, that only very little EV-DNA were colocalized with CD63^+^-sEVs (Fig. 4D), while most of them were not colocalized (Fig. 4[E-H]). To further understand the packaging of EV-DNA, we also performed dual-color SMLM imaging of HEK293T-CD63-GFP⁺-sEVs prior to their uptake by recipient cells. Interestingly, we observed no significant difference in the colocalization pattern of EV-DNA with CD63⁺-sEVs before and after internalization (Figure S4).

### Interaction of EV-DNA with cytoplasmic DsDNA sensor (cGAS)

As part of the innate immune defense, cytosolic accumulation of exogenous DNA from various sources—including pathogens and damaged host cells—activates the cyclic GMP-AMP synthase–stimulator of interferon genes (cGAS-STING) pathway [43]. Notably, this pathway has been implicated in diverse biological processes, including metastasis in breast cancer [13] and anti-tumor immunity mediated by cytotoxic T cells in response to EV-DNA from cancer cells [19]. Given the importance of cytosolic DNA sensing by cGAS, we aimed to investigate the spatial interaction between EV-DNA and cGAS in recipient cells using nanoscale imaging. Cells were subsequently stained using a rabbit anti-MB21D1 (cGAS) primary antibody and an Alexa Fluor 647 -conjugated goat anti-rabbit secondary antibody. EV-DNA was labeled via click chemistry using BODIPY-azide, following the protocol described earlier.

**Fig. 5:**
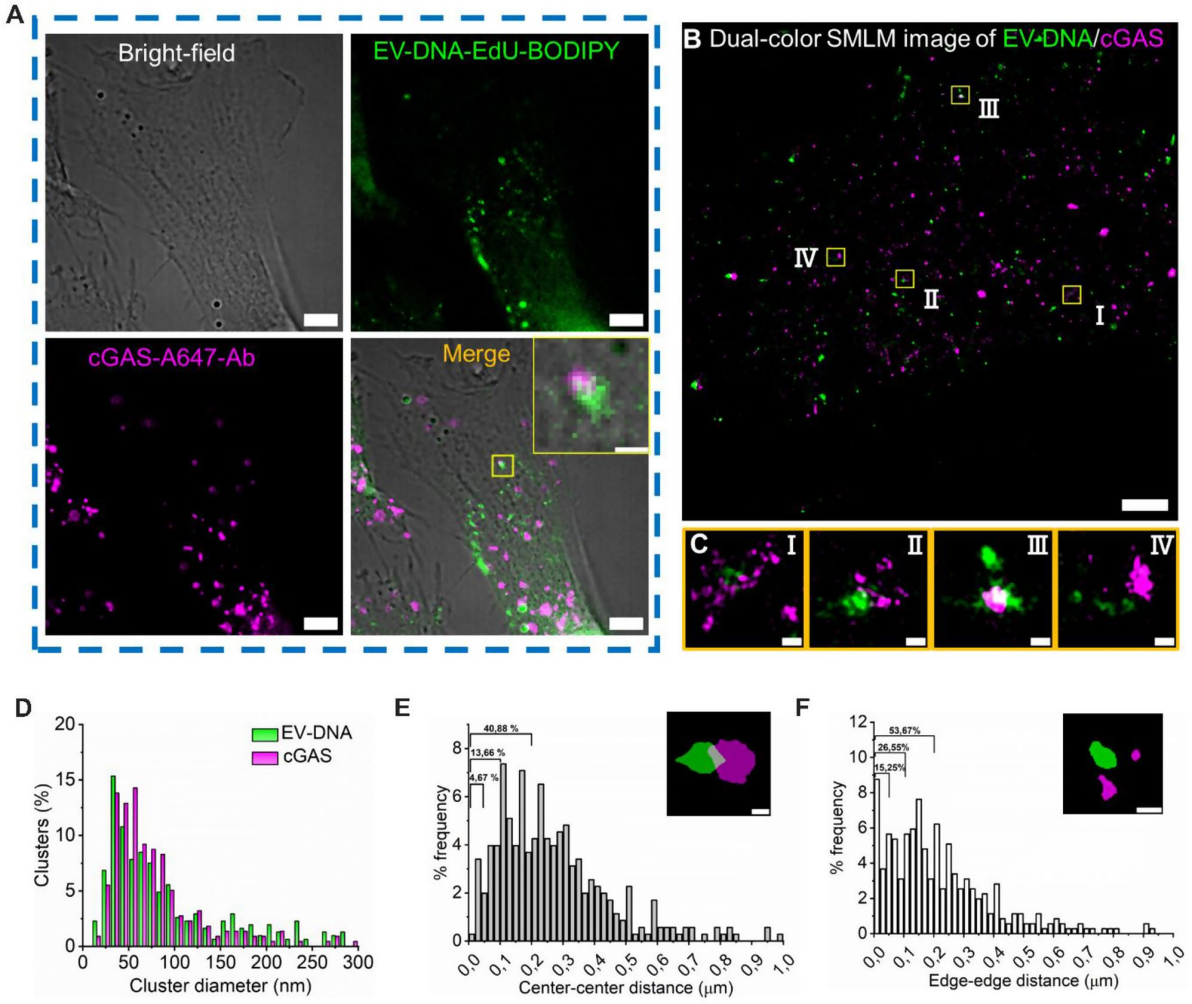
Interaction of EV-DNA and cGAS in HeLa cells. HeLa cells were treated with HEK293T-sEVs containing EdU-EV-DNA at a ratio of 50:1 (sEV particles per recipient cell). EV-DNA was detected via click chemistry using BODIPY-azide (green), and cGAS was labeled by immunofluorescence using a rabbit anti-MB21D1 primary antibody followed by an Alexa Fluor 647 -conjugated secondary antibody (purple). (**A**) Conventional fluorescence image showing cytoplasmic localization of EV-DNA and cGAS. The inset in the lower right shows the overlap of EV-DNA and cGAS. (**B**) Dual-color SMLM image of EV-DNA/cGAS in HeLa cells, providing nanoscale resolution. (**C**) Magnifications of four different areas marked with a yellow rectangle in B, displaying the interactions of EV-DNA (green) and cGAS (purple). (**D**) Voronoi cluster size distribution of EV-DNA (green) and cGAS (purple) clusters in B. (**E**) Center-center distance measurements between EV-DNA clusters and their nearest neighboring cGAS clusters. The graph shows the proportion of clusters falling within 0–50 nm, 0–100 nm, and 0–200 nm ranges. The inset shows the overlap of EV-DNA and cGAS clusters after segmentation, with white stars indicating the centers of EV-DNA and cGAS clusters. (**F**) Edge-edge minimum distance measurements between EV-DNA and cGAS clusters, with the same statistics applied in panel E. If EV-DNA and cGAS clusters overlap, the value will be zero. The inset shows the separation of EV-DNA and cGAS clusters after segmentation, and white stars illustrate the edges of EV-DNA and cGAS clusters, respectively. Scale bars: 5 μm in **A**, 2 μm in **B**, 1 μm in **A** (inset), 100 nm in **C**, **E** (inset), and **F** (inset)

In conventional wide-field fluorescence microscopy, apparent overlap between EV-DNA and cGAS was observed in the cytoplasm of recipient HeLa cells (Fig. 5A, inset); however, the resolution was insufficient to confirm molecular-level colocalization. To overcome this limitation, dual-color SMLM was employed (Fig. 5B). High-resolution imaging revealed that a subset of EV-DNA colocalized precisely with cGAS clusters (Fig. 5C, panels Ⅰ–Ⅲ), while others were clearly spatially separated (Fig. 5C, panel Ⅳ), suggesting that only a fraction of EV-DNA may directly interact with cGAS.

To quantify this interaction, we performed Voronoi-based cluster analysis, which showed that most cGAS (79%) and EV-DNA (70%) clusters had diameters smaller than 100 nm (Fig. 5D). Spatial relationship measurements between segmented clusters revealed that approximately 5% of EV-DNA–cGAS cluster pairs had a center–center distance below 50 nm, 14% below 100 nm, and 41% below 200 nm (Fig. 5E). Correspondingly, the edge–edge minimum distance was less than 50 nm for 15% of clusters, less than 100 nm for 27%, and less than 200 nm for 54% (Fig. 5F).

Considering the immunofluorescence labeling error caused by the size of both primary and secondary antibodies (~ 10 nm), the data indicate that approximately 15% of EV-DNA overlaps with cGAS in the cytoplasm indicating only partial potential interaction. However, it is important to note that this limited overlap may also reflect the transient or dynamic nature of EV-DNA–cGAS interactions, which static imaging cannot fully capture. Live-cell SMLM or correlative approaches may be needed to resolve these dynamics more comprehensively. Collectively, these findings underscore the utility of dual-color SMLM for studying the interaction of EV-DNA with subcellular structures in the recipient cells.

## Discussion

In this study, we successfully established a unique approach for nanoscale visualization of EV-DNA within recipient cells using single-molecule localization microscopy (SMLM). Notably, this is the first study to apply BODIPY, a green-emitting, buffer-independent blinking fluorophore, for SMLM of EV-DNA under physiological conditions (PBS buffer). By employing BODIPY, we overcame major limitations associated with conventional fluorophores and imaging buffers in multi-color SMLM, achieving high-resolution, artifact-free dual-color imaging of EV-DNA and subcellular components. This strategy enabled, for the first time, direct visualization of EV-DNA localization and interactions in recipient cells at nanometer resolution, offering new insights into its spatial organization and potential functional roles.

The newly optimized dual-color SMLM protocol, using BODIPY-azide for EV-DNA and Alexa Fluor 647 -conjugated nanobodies for GFP labeling, proved to be both reliable and adaptable for investigating EV-DNA biology at the nanoscale. Notably, the sequential labeling strategy we developed, which prevents copper-induced GFP quenching, represents a significant technical advancement for multi-color SMLM studies involving copper-dependent click chemistry, enabling highly accurate multi-target labeling with an exceptionally low linkage error (~ 2 nm). Together, these advances establish a broadly applicable workflow for nanoscale imaging of EV cargo interactions.

Among our major findings, we observed very low colocalization between EV-DNA and CD63⁺-sEVs, both before and after uptake by recipient cells. This suggests that EV-DNA is not predominantly associated with classical CD63⁺ vesicle populations but may instead reside in distinct subpopulations or loosely associated with vesicle membranes. These observations align with recent findings by Lázaro-Ibáñez et al., 2019, in which they have shown that small EV (sEV) subpopulations exhibit markedly different DNA profiles and topologies depending on their density [44]. Collectively, our data emphasize the need to further explore EV subtypes involved in DNA transport and their functional relevance.

We also investigated the interaction between EV-DNA and the cytosolic DNA sensor cGAS. Although some EV-DNA clusters were observed in close proximity to cGAS, the overall colocalization remained low (~ 15% based on edge-to-edge analysis). This limited overlap suggests that only a subset of EV-DNA may participate in cGAS-mediated signaling. These observations are consistent with prior studies reporting EV-DNA involvement in innate immune activation [16, 19, 20, 45] but also point to the possibility of transient or spatially restricted interactions. However, spatial proximity alone does not demonstrate functional interaction, and confirming biological relevance will require additional functional studies, such as STING activation assays. Future live-cell SMLM and correlative imaging approaches will be crucial for fully capturing these dynamics in real time.

Our results open several promising avenues for future research. The development of red and near-infrared buffer-independent blinking fluorophores will enable more advanced multi-color live-cell SMLM imaging [46, 47]. The integration of copper-free click chemistry could further improve labeling efficiency and facilitate real-time tracking of EV-DNA interactions in living systems. Additionally, expanding the repertoire of EV markers beyond CD63 (e.g., CD9, CD81) will help further resolve EV heterogeneity and DNA cargo specificity.

While our work advances the field of EV-DNA imaging, some limitations must be acknowledged. The use of fixed cells restricts observation of dynamic cargo transfer and interactions; employing buffer-independent fluorophores, such as BODIPY, in live-cell SMLM could address this limitation. Additionally, the biological significance of EV-DNA-cGAS interactions needs validation through functional assays, such as measuring interferon signaling. Furthermore, as this study used 2D SMLM, distance measurements only capture lateral (x-y) relationships, leaving z-axis interactions unresolved. Future two-color 3D SMLM would allow comprehensive 3D mapping of EV-DNA dynamics within recipient cells.

Taken together, our results establish SMLM as a powerful tool for investigating EV-DNA packaging, delivery, and subcellular interactions within recipient cells at nanometer resolution. This methodology provides a foundation for exploring the molecular mechanisms underlying EV-DNA function in health and disease. Advancements in live-cell multi-color SMLM and novel fluorophores will ultimately enable the real-time tracking of EV-DNA and thereby, deepening our understanding of its role in intercellular communication and disease progression.

## Conclusions

This study introduces a unique SMLM-based approach using BODIPY, a buffer-independent blinking fluorophore, for nanoscale imaging of EV-DNA in recipient cells. By combining click chemistry for EV-DNA with nanobody-based labeling of CD63-GFP⁺-sEVs, we established a sequential protocol that minimizes linkage error and prevents copper-induced GFP quenching. Dual-color SMLM imaging revealed limited colocalization between EV-DNA and CD63⁺-sEVs, suggesting EV-DNA may associate with distinct vesicle populations. We also observed limited interaction between EV-DNA and the cytoplasmic DNA sensor cGAS, indicating a potential but selective role in immune sensing. Together, these findings demonstrate the power of this dual-color imaging strategy for dissecting EV-DNA dynamics and lay the groundwork for future live-cell, multi-color SMLM studies of EV-mediated communication in health and disease.

## Supplementary Information

Below is the link to the electronic supplementary material.

Supplementary Material 1

Supplementary Material 2

## Data Availability

The datasets used and/or analyzed during the current study are available from the corresponding authors on reasonable request.

## Abbreviations

SMLM: Single-molecule localization microscopy
EdU: 5-ethynyl-2’-deoxyuridine
F-ara-EdU: (2′S)-2′-Deoxy-2′-fluoro-5-ethynyluridine
BODIPY: Boron-dipyrromethene
Nb: Nanobody
sEVs: Small extracellular vesicles
EV-DNA: DNA associated with small extracellular vesicles
cGAS: Cyclic GMP-AMP synthase
STING: Stimulator of interferon genes
NTA: Nanoparticle-tracking analyzer
TEM: Transmission electron microscopy
DMEM: Dulbecco’s Modified Eagle’s Medium
FBS: Fetal bovine serum
P/S: Penicillin/ Streptomycin
TSU: Tangential Flow Filtration + Size Exclusion Chromatography + Ultrafiltration
GFP: Green fluorescent protein
BSA: Bovine serum albumin
dsDNase: Double stranded DNase
