# Supplementary figures and images for: Single-molecule localization microscopy imaging of extracellular vesicle DNA in recipient cells
Source: J Transl Med. 2026 Jan 3;24:130. doi: 10.1186/s12967-025-07563-3 (PMC12866493; doi:10.1186/s12967-025-07563-3)

## Original Blots

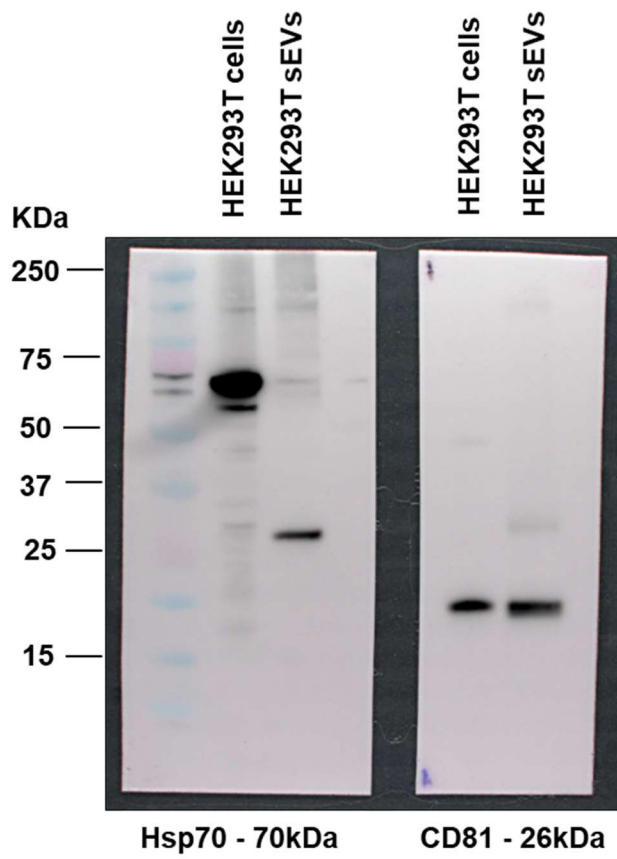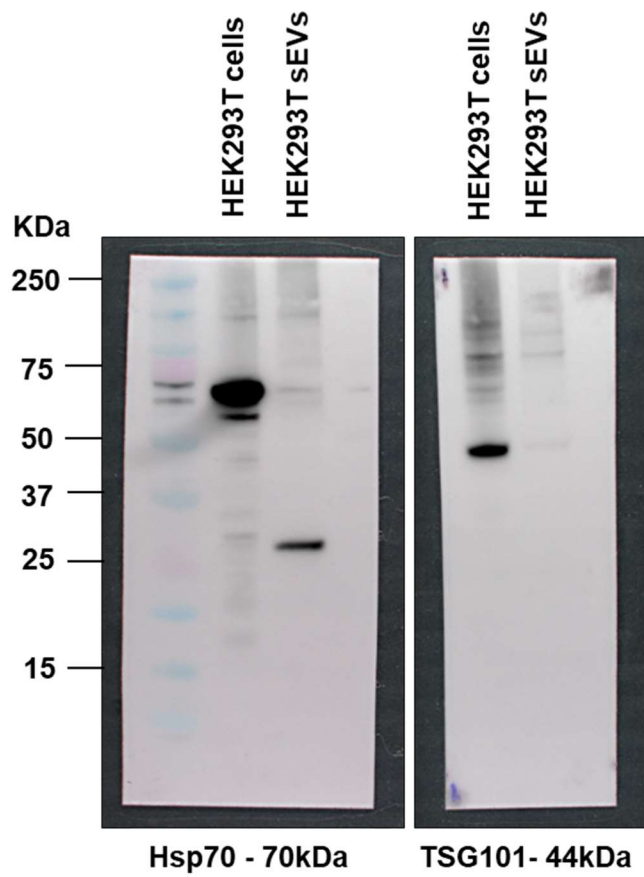

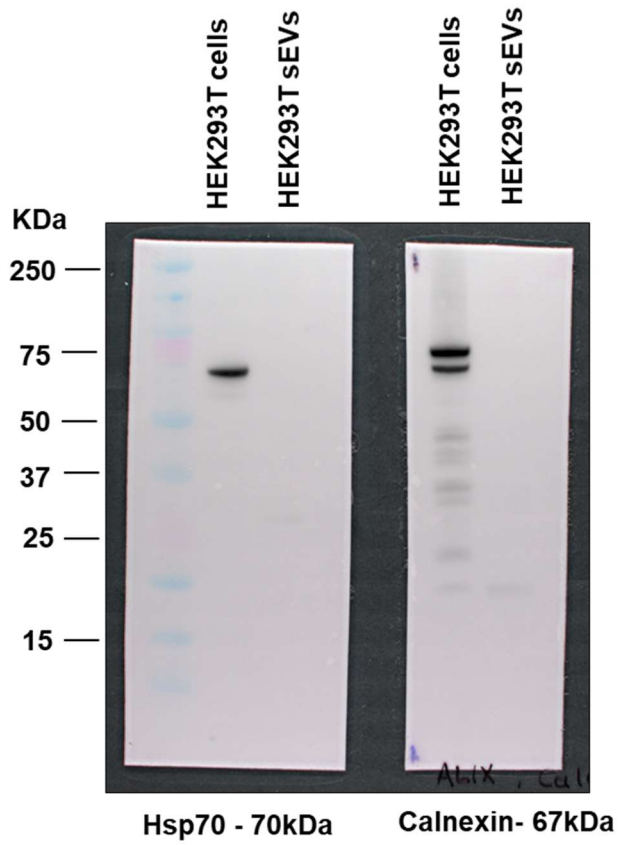

Supplement: Supplementary file 1 — Supplementary Material 1 [file 12967_2025_7563_MOESM1_ESM.pdf]
