# Supplementary material for: Single-molecule localization microscopy imaging of extracellular vesicle DNA in recipient cells
Source: J Transl Med. 2026 Jan 3;24:130. doi: 10.1186/s12967-025-07563-3 (PMC12866493; doi:10.1186/s12967-025-07563-3)
Supplement: Supplementary file 2 — Supplementary Material 2 [file 12967_2025_7563_MOESM2_ESM.docx]

**Supplementary Figures**

**Single-Molecule Localization Microscopy Imaging of Extracellular Vesicle DNA in Recipient Cells**

Xingfu Zhu^#[1]^, Venkatesh Kumar Chetty^#[2,3]^, Jamal Ghanam^[2,3]^, Anisa Hila^[2,4]^, Qiqi Yang^[1]^, Hilmar Strickfaden^[5]^, Mischa Bonn^[1]^, Christoph Cremer^[1,6]^, Peter F. Hoyer^[7]^, Xiaomin Liu*^[1]^, and Basant Kumar Thakur*^[2,4,8]^

1. Department of Molecular Spectroscopy, Max Planck Institute for Polymer Research, Ackermannweg 10, 55128 Mainz, Germany.
2. Department of Pediatrics III, University Hospital Essen, Hufelandstrasse 55, 45147 Essen, Germany.
3. Department of Gastroenterology, Hepatology and Transplant Medicine, Medical Faculty, University Hospital Essen, Hufelandstrasse 55, 45147 Essen, Germany.
4. Department of General, Visceral, Vascular and Transplant Surgery, Medical Faculty, University Hospital Essen, Hufelandstrasse 55, 45147 Essen, Germany.
5. Cell Imaging Centre, Faculty of Medicine and Dentistry, B-120 Katz Group Centre, University of Alberta, Edmonton, AB, T6G 2T9, Canada.
6. Institute of Molecular Biology (IMB), Ackermannweg 4, 55128 Mainz, Germany.
7. Department of Pediatrics II, University Hospital Essen, Hufelandstrasse 55, 45147, Essen, Germany.
8. European Liquid Biopsy Society (ELBS), Hamburg, Germany.

^#^Xingfu Zhu and Venkatesh Kumar Chetty, contributed equally to this work.

*Correspondence- [basant-kumar.thakur@uk-essen.de](mailto:basant-kumar.thakur@uk-essen.de) (BKT) and [liuxiaomin@mpip-mainz.mpg.de](mailto:liuxiaomin@mpip-mainz.mpg.de) (XL)


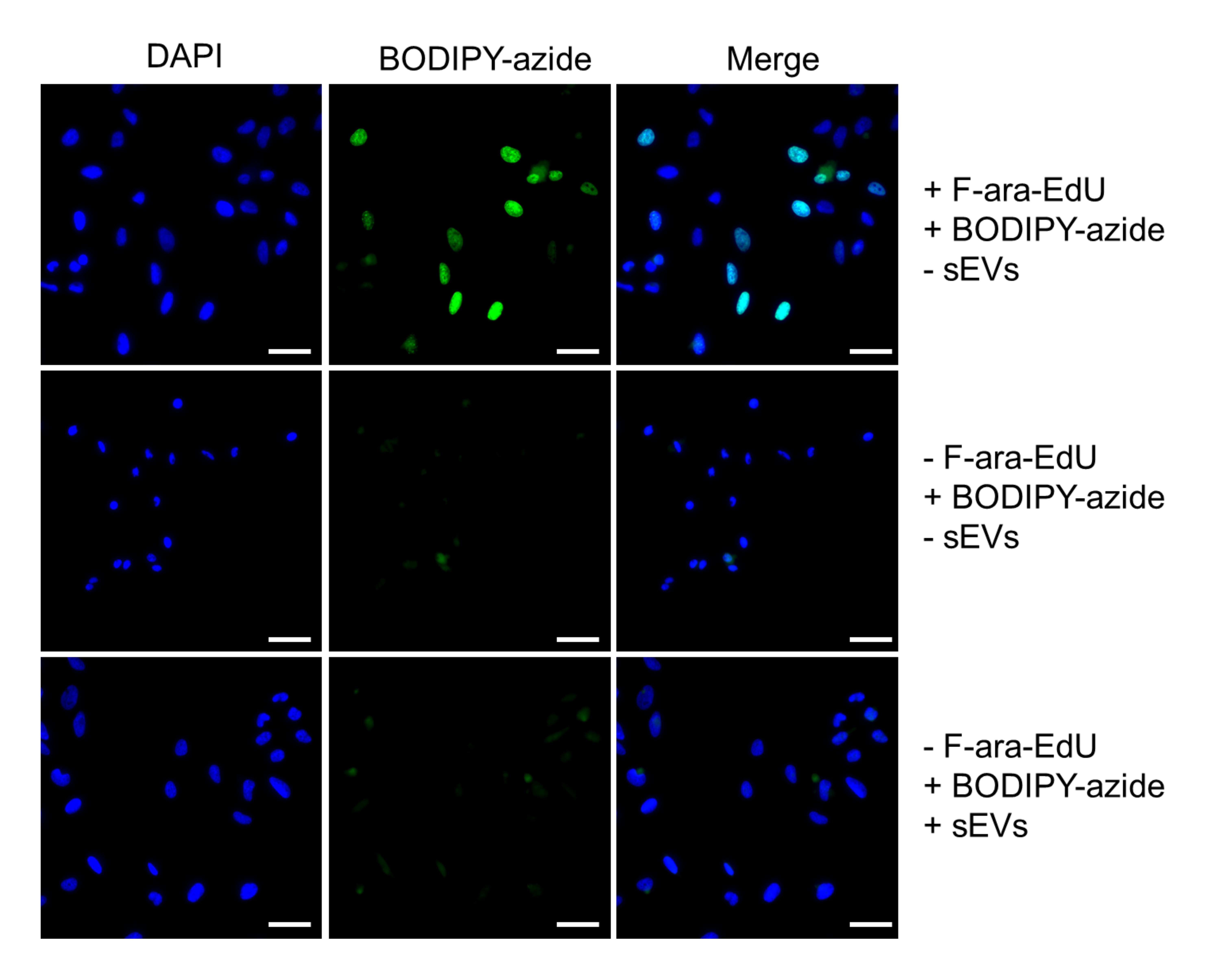


**Supplementary Figure S1.** Representative wide-field images of HeLa cells treated with F-ara-EdU and HEK293T sEVs in PBS. First panel displays HeLa cells treated with F-ara-EdU and the newly synthesized DNA labeled with EdU was detected using click reaction via BODIPY-azide (green). Middle panel shows untreated HeLa cells, which were still subjected to click reaction with BODIPY-azide to calculate the BODIPY background signal. Bottom panel displays HeLa cells treated with HEK293T sEVs that do not contain EV-DNA-EdU and were subjected to the same BODIPY-azide click reaction to evaluate the false positive signal of EV-DNA. Laser intensity and exposure time were the same for all three samples. DAPI and BODIPY-azide were excited using 405 nm and 475 nm lasers respectively. Scale bars: 50 µm.

**Supplementary Figure S2.** Representative wide-field images of U2OS cells that express GFP on the nuclear pore complex (Nup96), treated with and without copper ion before immunostaining. Top panel displays untreated U20S cells with Nup96-GFP and the inset is corresponding bright-field image. Middle panel shows U2OS cells with Nup96-GFP with no copper treatment prior to labeling by Alexa647-anti-GFP-nanobody (Alexa647-Nb). Bottom panel displays U2OS cells with Nup96-GFP that was treated with copper ions prior to immunostaining (Alexa647-Nb), and the inset is the corresponding bright-field image. Brightness scale of images is shown on the upper right corner of each image. Scale bars: 5 µm.


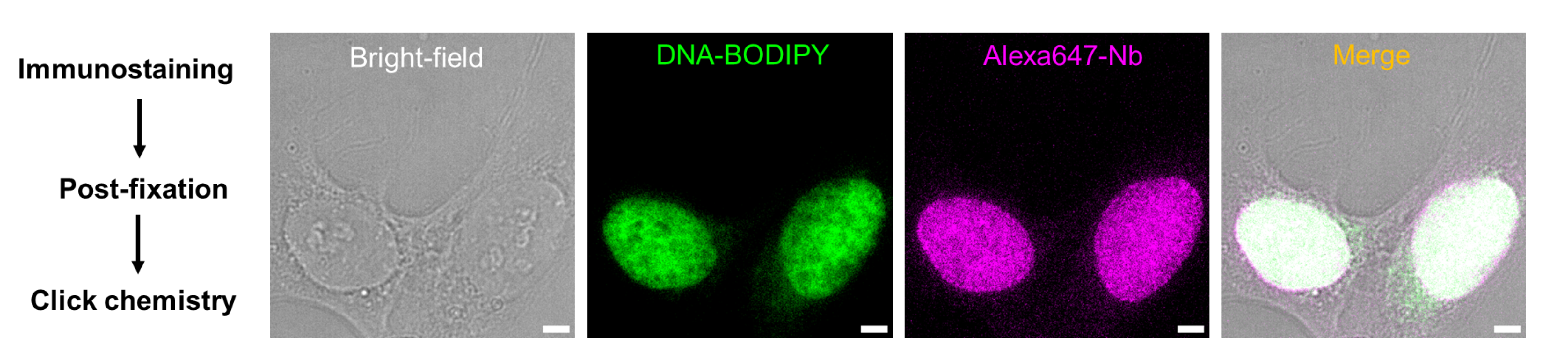


**Supplementary Figure S3.** Simultaneous labeling of nascent DNA and Nup96-GFP in U2OS cells that express GFP on nuclear pore complex (Nup96). U2OS-Nup96-GFP cells were incubated with F-ara-EdU (20 uM) for 3 hours. Nup96-GFP was first labelled with Alexa647-anti-GFP-nanobody (Alexa647-Nb) by immunostaining and then post fixation, F-ara-EdU labeled nascent DNA was detected using BODIPY-azide via click chemistry. Scale bars: 5 µm.


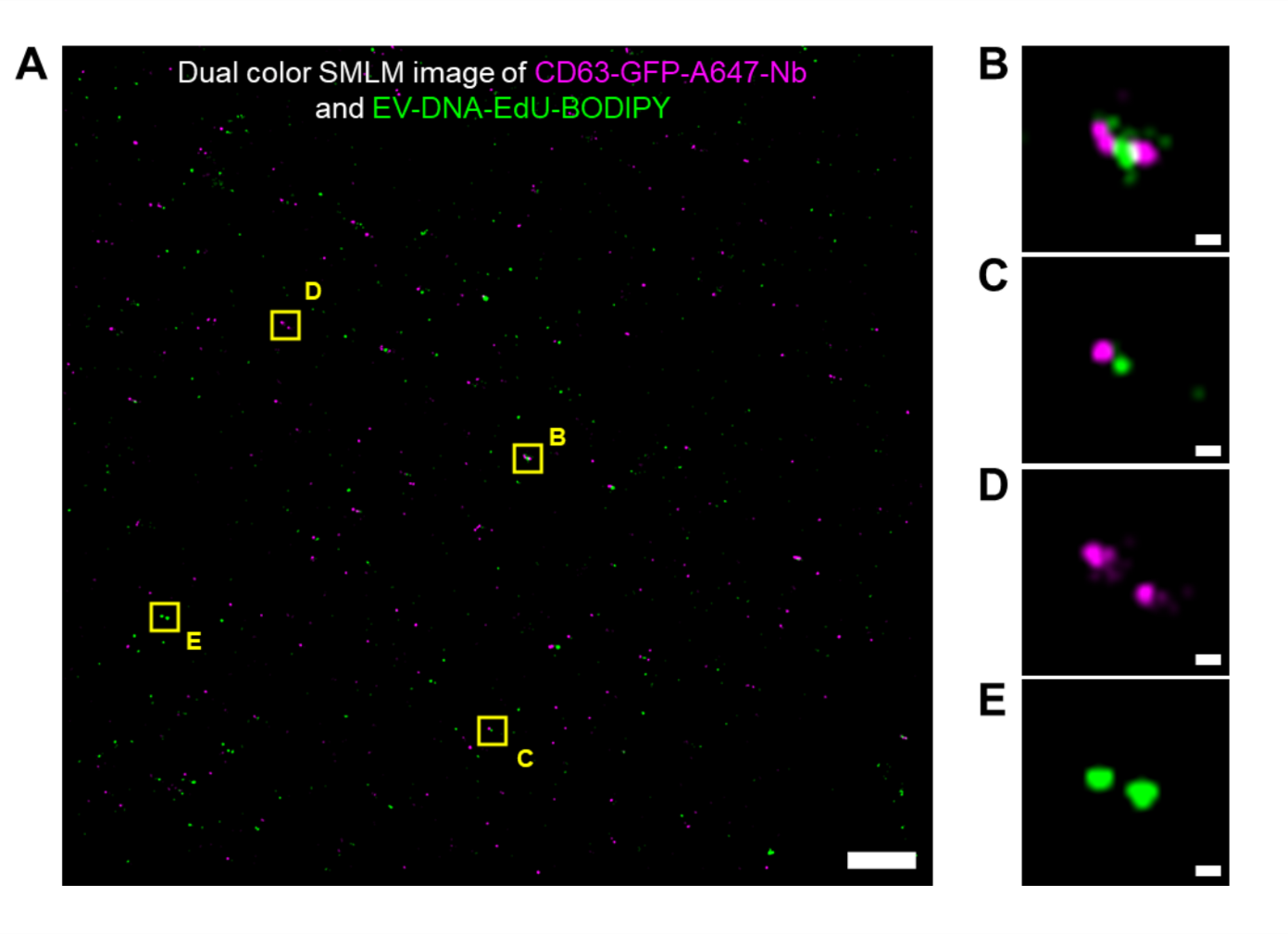


**Supplementary Figure S4.** Dual-color SMLM imaging of A) HEK293T-CD63-GFP^+^-sEVs containing EV-DNA-EdU. EV-DNA-EdU was labeled by BODIPY-azide via click chemistry and CD63-GFP^+^-sEVs was labeled by A647-Nb. (B-E) Magnification of yellow rectangle marked area in A. The imaging was performed in a special blinking buffer and an oxygen scavenging system. Scale bars: 2 µm in A, 100 nm in B, C, D and E.


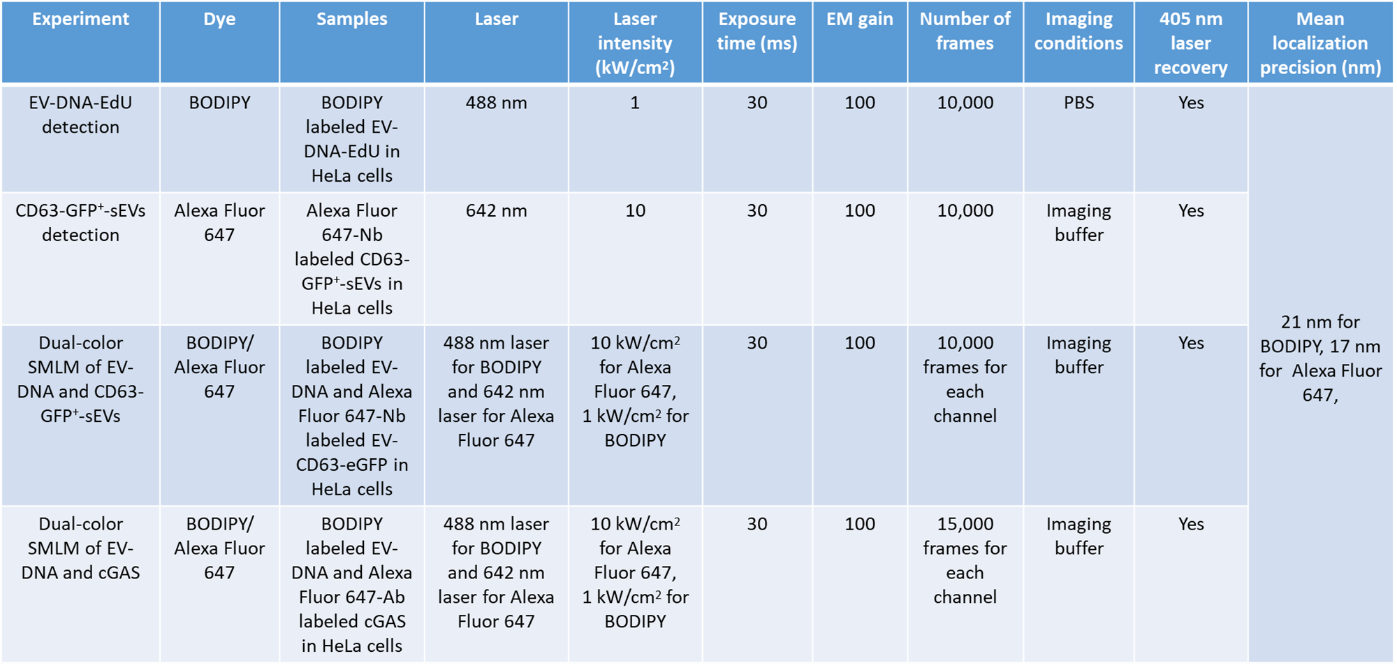


**Supplementary Table S1.** SMLM imaging parameters. Imaging buffer composition: 50 mM Tris (pH 8.0), and an oxygen scavenging system (0.5 mg/ml glucose oxidase, 40 µg/ml catalase, and 10% (w/v) glucose) and 143 mM βME. Nb-nanobody, Ab-antibody.
